# Plasma levels of BCMA-positive extracellular vesicles correlate to response and side effects in myeloma patients treated with belantamab-mafodotin

**DOI:** 10.18632/oncotarget.28538

**Published:** 2023-12-01

**Authors:** Carsten Springer, Jürgen Krauter, Arne Trummer

**Affiliations:** ^1^Department of Hematology and Oncology, Städtisches Klinikum Braunschweig, Braunschweig, Germany; ^2^Department of Hematology, Oncology and Palliative Care, Heidekreis-Klinikum, Walsrode, Germany

**Keywords:** myeloma, b cell maturation antigen, extracellular vesicles, belantamab-mafodotin, eryptosis

## Abstract

In myeloma patients, high levels of soluble BCMA (sBCMA) can limit the efficacy of BCMA-directed therapies. Belantamab-mafodotin is a BCMA antibody-drug conjugate and shows good overall response rates in heavily pretreated patients but progression-free survival data are poor. As the drug induces apoptosis, we hypothesized that sBCMA includes extracellular vesicles (EV) and thus evaluated numbers of BCMA-EV before and during belantamab therapy in 10 myeloma patients. BCMA-EV were significantly higher in patients prior to Belantamab (median: 3227/μl; *p* = .013) than in other myeloma patients before therapy (*n* = 10; 1082/μl) or healthy volunteers (*n* = 10; 980/μl). During therapy, BCMA-EV showed a significant increase to a maximum of 8292/μl (*p* = .028). Maximal changes in BCMA-EV (Δmax = BCMA-EV at C1/maximal BCMA-EV) showed a strong inverse, logarithmic correlation (r = −.950; *p* < .001) with FLC ratio changes (Δmax = FLC ratio at C1/minimal FLC ratio) and BCMA-EV peaks often preceded FLC progression. Correlating increase of LDH and BCMA-EV levels, together with clinical symptoms, point to a mafodotin-induced eryptosis. In summary, BCMA-EV are a part of sBCMA, peak levels precede progression, and their measurement might be helpful in identifying resistance mechanisms and side effects of BCMA targeted therapies.

## INTRODUCTION

Multiple myeloma (MM) progression strongly depends on crosstalk between tumor cells and cells of the surrounding microenvironment creating a tumor supportive niche. Extracellular vesicles (EV) play a key role in the modification of this niche through their surface molecules as well as their biologically active cargo [1, 2].

Belantamab-mafodotin, an antibody-drug conjugate (ADC) targeting B-cell maturation antigen (BCMA), shows good overall response rates of at least 40% in heavily pretreated myeloma patients but responses are mostly short-lived with a median progression-free survival (PFS) of around 3 to 4 months in real-world cohorts [3–6] and no PFS benefit compared to pomalidomide/dexamethasone in the randomized DREAMM-3 trial [7].

It has been demonstrated that high serum levels of soluble BCMA (sBCMA) can inhibit anti-BCMA directed therapies by competitive binding [8]. BCMA is actively cleaved from the myeloma cell surface by the ubiquitous multisubunit γ-secretase (GS) complex. On one side, this leads to reduced ligand density on myeloma cells, and on the other side, sufficient sBCMA can accumulate in the bone marrow and inhibit BCMA antibody binding or CAR T-cell recognition of myeloma cells. Taken together, these changes limit the efficacy of BCMA-directed therapy [9].

Belantamab-mafodotin induces cell death due to caspase 3-dependent apoptosis and membrane blebbing thereby releasing extracellular vesicles (EV). We were therefore interested if “soluble BCMA” in blood plasma includes EV carrying BCMA or other myeloma antigens and if these BCMA-EV levels show a significant change during therapy with belantamab-mafodotin.

## RESULTS

### Patient characteristics

10 consecutive myeloma patients (5 female, 5 male) with clinical indication for treatment with Belantamab were included. Myeloma types were IgG (*n* = 4), IgA (*n* = 3) and light chain (*n* = 3). Median age was 73.0 years (range: 48–79) and patients had received a median of 6 prior therapies (4–13). Median time from initial diagnosis to start of Belantamab was 6.2 years (1.3–13.9).

Myeloma patients in the control group (5 female, 5 male) had a median age of 68.0 years (63–81) and 0 to 6 prior therapies with 6 patients having their primary diagnosis of myeloma. Healthy volunteers (8 female, 2 male) had a median age of 47 years (39–61).

### Patient outcomes

Patients received a median of 2.0 (1–8) courses of Belantamab 2.5 mg/kg every 3 weeks, corresponding to a median duration of treatment (DOT) of 2.2 months (0.7–9.2). Overall response rate (ORR) was 50% with 2 complete remissions, 1 partial remission and 2 minor responses. One patient had a stable disease and 4 patients a progressive disease. Seven patients (70%) discontinued therapy due to progression and 3 (30%) due to toxicity (keratopathy, sepsis), of which one was able to continue therapy anew with high dose melphalan and autologous stem cell transplantation. Altogether 7 patients (70%) received at least one subsequent therapy.

Median progression-free survival (PFS) from start of Belantamab therapy was 2.6 months (95% CI: 0.5–4.7 months) until therapy had to be stopped due to progression (70%) or toxicity (30%). Median overall survival was 7.8 months (95% CI: 0.5–15.4 months). In univariate Cox regression analysis, only the change in FLC ratio (Δmax FLC ratio) was associated with PFS.

### Total EV and BCMA-EV

**Figure 1:**
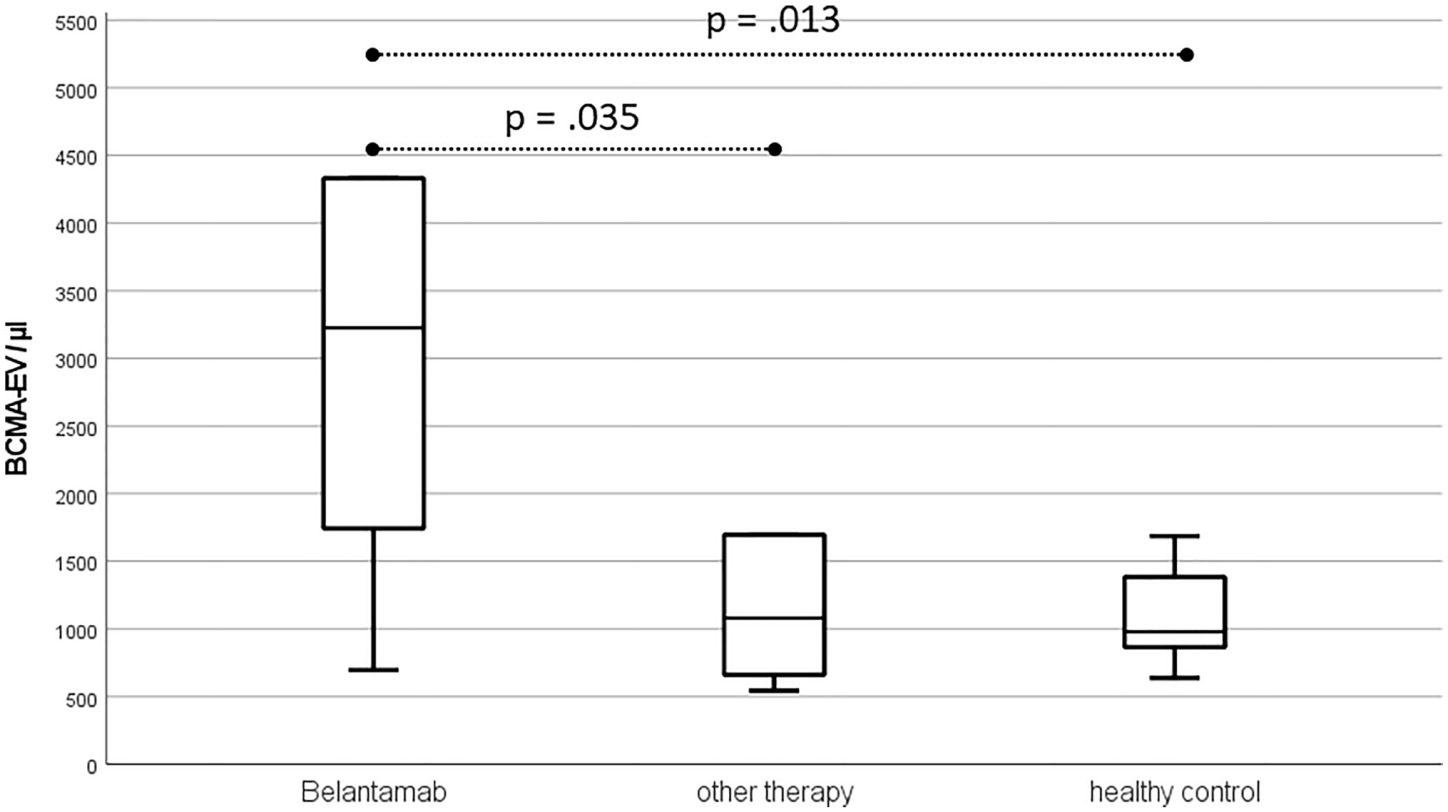
Boxplots for BCMA-EV prior to start of therapy excluding outliers.

The median number of total EV (Annexin V positive EV) before start of therapy was 7559/μl (1554–223825/μl) for Belantamab patients. This was significantly higher than for the myeloma control group before therapy (3133/μl; 833–14413/μl; *p* = .043) and for healthy donors (3259/μl; 1487–9553/μl; *p* = .028). Accordingly, also BCMA-EV were significantly higher in patients prior to Belantamab (3227/μl; 695–203024/μl) than in other myeloma patients before therapy (1082/μl; 544–8548/μl; *p* = .035) or healthy volunteers (980/μl; 638–2468/μl, *p* = .013) (Figure 1).

During therapy, EV showed a significant increase during Belantamab therapy to a maximum of median total EV of 38919/μl (2604–223825/μl, *p* = .028) and to a maximum of median BCMA-EV of 8292/μl (1170–203024/μl; *p* = .028).

### Correlation of EV with treatment and response

**Figure 2:**
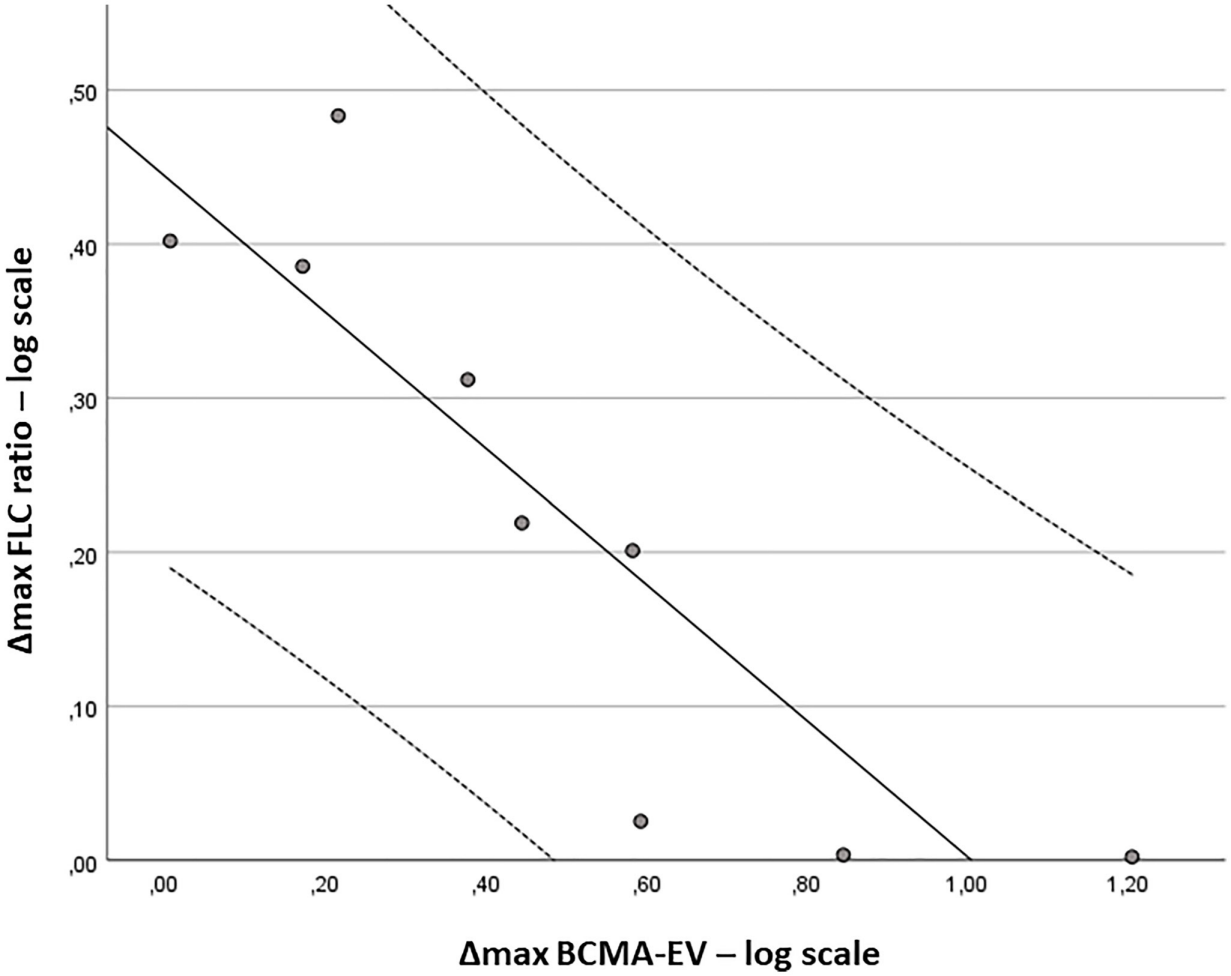
Correlation of free light chain ratio response to BCMA-EV changes. Shown are regression curve (R^2^ = 0.78) with 95% confidence intervals. Δmax FLC ratio = FLC ratio at C1/FLC ratio at Cmin; Δmax BCMA-EV = BCMA-EV per μl at C1/BCMA-EV per μl at Cmax.

**Figure 3:**
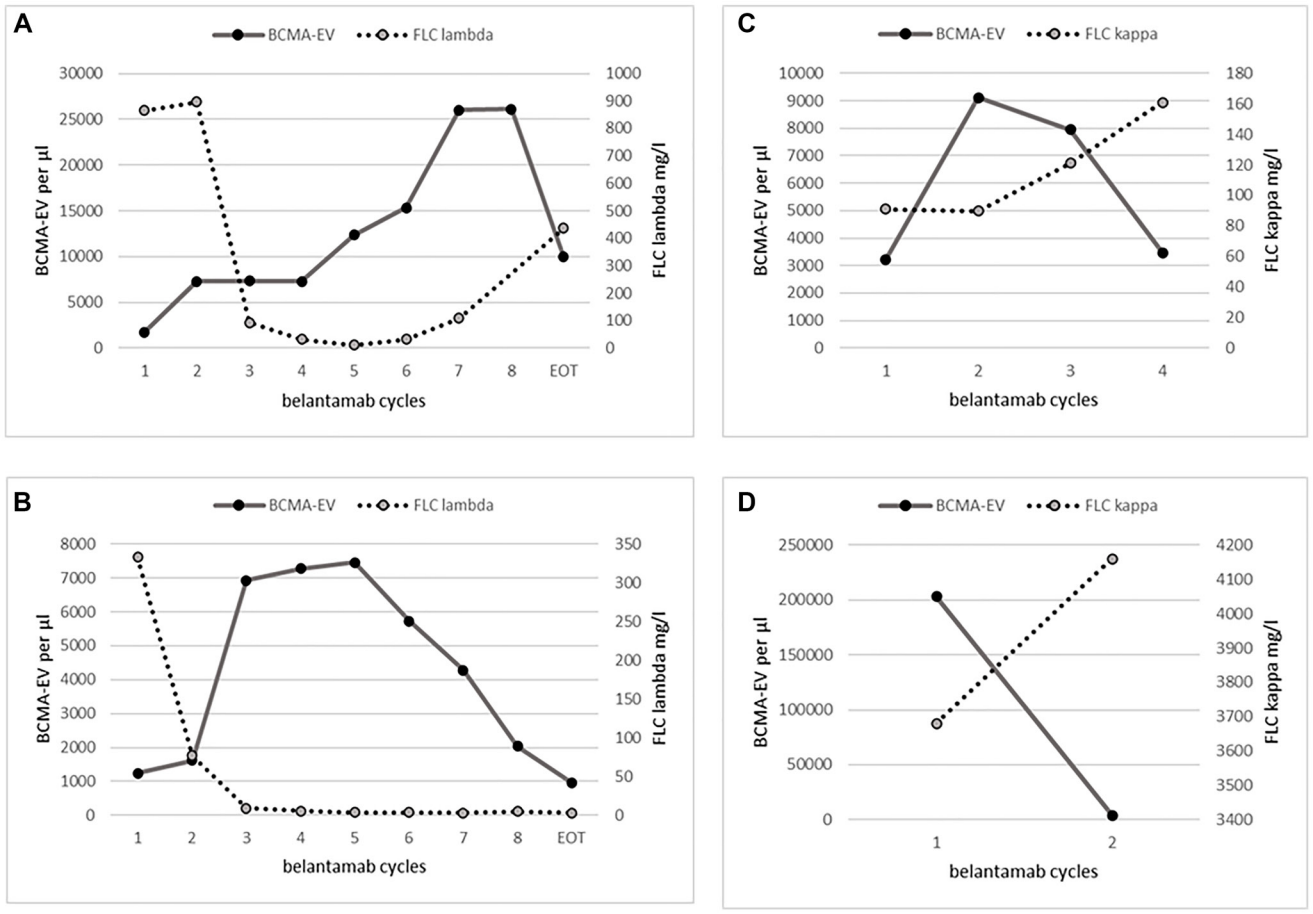
Patient examples of inverse correlation of BCMA and FLC changes during course of therapy. (**A**) Shows a patient with complete response but continuous rise in BCMA-EV levels and finally FLC progression after reaching maximum BCMA-EV levels. (**B**) Shows a patient with ongoing complete remission who died of sepsis. (**C**) Shows a patient who achieved stable disease in FLC after one cycle but already reached maximum BCMA-EV levels and then showed progression. (**D**) Shows a patient whose initial BCMA-EV levels were extremely high and who was refractory to Belantamab therapy.

FLC ratio changes (Δmax FLC ratio = FLC ratio at C1/minimal FLC ratio) showed a strong inverse, non-linear correlation with maximal changes in BCMA-EV counts (Δmax BCMA-EV = BCMA-EV at C1/maximal BCMA-EV; r = −.950; *p* < .001) as shown in logarithmic scale in Figure 2. However, the maximal changes in BCMA-EV counts had only a weak correlation to the best response (r = .673; *p* = .033) and did not correlate to the number of Belantamab cycles (r = .565) or the treatment duration (r = .583) as high BCMA-EV counts often preceded FLC progression as demonstrated in four patient examples in Figure 3.

Patients with a strong increase in BCMA-EV after first cycle (ΔC1/C2 BCMA-EV = BCMA-EV at C1/BCMA-EV at C2) were more likely to have a strong increase of LDH values after the first cycle (ΔC1/C2 LDH = LDH at C1/LDH at C2) (r = .709; *p* < .022). To further elucidate the cause of the LDH increase, we measured CD235a-positive, red blood cell (RBC) derived EV in 5 samples of 2 patients who had developed a classical constellation of hemolysis with reduced haptoglobin and significant fatigue, but had negative coombs tests, no reduction in hemoglobin values and no increase in bilirubin. We found a moderate increase in RBC-EVs in both patients (median 1641/μl, range: 586–2374/μl) which paralleled rising LDH values.

### Other cell antigens for detection of myeloma EVs

To evaluate if other antigens might be preferable to BCMA for the detection of myeloma EVs, we evaluated 15 samples from 5 of the 10 patients treated with Belantamab (cycles 1 to 3) and quantified EVs with the following antigens: CD23, CD27, CD38, CD56 and CD138. As shown in Table 1, the numbers were many times lower than for BCMA-EV levels.

**Table 1:** EV measurement using other possible myeloma antigens (median and range of 5 patients from cycle 1 to 3)

	Cycle 1	Cycle 2	Cycle 3
**BCMA**	5839	28048	10749
	(2040–46982)	(7356–59344)	(6209–40689)
**CD23**	56	40	177
	(39–247)	(21–66)	(52–411)
**CD27**	128	160	164
	(29–332)	(108–251)	(125–216)
**CD38**	397	895	449
	(222–1283)	(337–1374)	(323–1296)
**CD56**	212	272	208
	(106–413)	(148–384)	(201–1448)
**CD138**	336	626	262
	(237–1020)	(328–1026)	(240–1630)

## DISCUSSION

To the best of our knowledge, we demonstrate for the first time that BCMA-positive extracellular vesicles can be found in blood plasma from myeloma patients and that BCMA expression on EV is 10 to 100 times higher than that of other well-known antigens of myeloma cells.

Thus, the so far used term “soluble BCMA (sBCMA)” includes unbound BCMA and EV-bound BCMA in plasma [10, 11]. Similar to sBCMA [10, 12], we found low levels of BCMA-EV in healthy donors and a correlation of BCMA-EV with disease status as BCMA-EV levels in heavily pretreated patients were significantly higher than in myeloma patients without prior treatment or in early course of therapy.

However, after logarithmic transformation sBCMA levels have shown a linearized positive correlation to the percentage of bone marrow plasma cells and have also paralleled the course of monoclonal protein or FLC levels, thereby predicting outcome [11]. In contrast, we have found an inverse logarithmic correlation between BCMA-EV changes and FLC ratio changes. In addition, BCMA-EV levels often increased directly after start of belantamab therapy and frequently peaked just before FLC levels were rising again. This phenomenon might be well explained by the inhibitory effect of high sBCMA levels on anti-BCMA directed therapies due to competitive binding [8], limiting the efficacy of BCMA-directed therapies [9]. This is reflected by the FDA request for withdrawal of belantamab due to negative results of the DREAMM-3 trial in which belantamab did not improve PFS compared to pomalidomid/dexamethasone despite an ORR of 41%. Accordingly, real-world data also show an ORR of 40–45% but only a PFS of 3 to 4.7 months [3–6]. These data are very similar to the outcome in our cohort with an ORR of 50% but a very short PFS of only 2.6 months. The rather short duration of treatment is in fact a limitation of our study and might be explained by the high number of prior therapies (4–13) and already high BCMA-EV levels prior to belantamab. Another clinically important implication of competitive sBCMA binding is most likely the improvement of BCMA-CAR-T-cell efficacy when applied >6 months after other BCMA targeted therapies (ORR 82.8%) compared to <6 months (ORR 60%) [13].

The release of sBCMA from the myeloma cell membrane is induced by the γ-secretase (GS) complex and also leads to reduced ligand density [9]. Inhibition of GS with an oral small-molecule inhibitor (GSI) leads to a thirtyfold increase of BCMA expression on myeloma cells and the combination of GSI with concurrent BCMA targeted therapy is evaluated in a clinical trial (#NCT03502577). Clinical efficacy and tolerability of another GSI, nirogacestat, has already been evaluated in a phase III study for desmoid tumors [14].

Belantamab is an antibody conjugated to mafodotin (monomethyl auristatin, MMAF). After internalization, belantamab-mafodotin induces cell death through cell cycle arrest and caspase 3-dependent apoptosis and membrane blebbing [15, 16]. Furthermore, monomethyl auristatin inactivates the Akt/mTOR pathway [17] and a classical side effect of mTOR inhibitors like rapamycin and temsirolimus is anemia due to eryptosis with erythrocyte shrinkage, membrane scrambling and release of phospholipids [18]. In contrast to hemolysis, the plasma membrane of eryptotic cells remains intact and hemoglobin is mostly retained in the cytoplasm [19]. Both, caspase-3 induced EV generation and eryptosis might well explain the observed increase in LDH and minor signs of hemolysis (fatigue, reduced haptoglobin) as well as the positive correlation of LDH and BCMA-EV after start of belantamab-mafodotin. In case of overt hemolysis, RBC-EV should be markedly increased [20] but in our 2 patients with relevant LDH elevation we found only a moderate increase of RBC-EV. Macropinocytosis has been implicated as a mechanism for the development of thrombocytopenia and keratopathy, adverse events commonly reported under treatment with belantamab mafodotin and other MMAF-containing ADCs [21] and, thus, it appears likely that eryptosis is also based on the same mechanism.

In conclusion, we have shown that belantamab mafodotin induces BCMA-EV which inversely correlate to FLC levels and peak BCMA-EV levels often precede myeloma progression. Being a part of sBCMA, BCMA-EV most likely contribute to the limited efficacy of BCMA-directed therapies by competitive binding and their correlation to LDH most likely reflects the mafodotin-induced phenomenon of eryptosis. Measurement of BCMA-EV can thus help in identifying resistance mechanisms and side effects of BCMA targeted therapies in future studies.

## MATERIALS AND METHODS

### Patients and study design

From May 2020 until May 2022, peripheral blood (PB) samples were taken from 10 consecutive myeloma patients undergoing treatment with belantamab-mafodotin. Samples were taken before every Belantamab infusion and, if possible, after progression at the start of the next treatment line.

As controls, PB samples were obtained from both 10 other myeloma patients at the time point of initial diagnosis or during a therapy protocol without BCMA-directed therapy as well as from 10 healthy volunteers.

### Blood sampling and preparation of platelet-poor plasma

PB was drawn from patients directly into commercially available buffered sodium citrate tubes (citrate 0.129 mol/l; pH 5.5; Sarstedt, Germany) and was processed within 4 hours. For preparation of platelet-rich plasma (PRP), samples were centrifuged at 200 g for 8 min. Then, 1 ml of supernatant was additionally centrifuged at 1500 g for 20 min for preparation of for platelet-poor plasma (PPP), both at room temperature. Finally, 500 μl supernatant was frozen as PPP at −80°C until further processing. Thawing for further processing was done at room temperature.

### Reagents and monoclonal antibodies

Antibodies and fluorochromes to the following antigens were used:

Annexin V-Cy5 (BD Biosciences, Germany), IgG1-VioBright-FITC and CD269/BCMA-VioBright-FITC (Miltenyi Biotec, Germany), IgG1-FITC and CD23-FITC (Beckmann Coulter, Germany), CD27-PC5 (BioLegend, Germany), IgG1-PE and CD38-PE (Beckmann Coulter), IgG1-PC5 and CD56-PC5 (Beckmann Coulter), IgG1-PacificBlue and CD138-PacificBlue (Biolegend), CD235a-PE (Beckmann Coulter).

Annexin V binding buffer 20× (Milteny Biotec), Aqua dest. and TruCount Tubes^®^ (BD Biosciences).

### Antibody labelling of EV

Samples were kept on ice during the entire process to avoid coagulation. 50 μl of PPP samples were resuspended either in 50 μl Annexin binding buffer or control buffer and EVs were labeled with 1,5 μl Annexin V-Cy5 and 5 μl of each primary antibody or isotype control. After thorough mixing, samples were incubated for 30 min at 4°C in the dark. Finally, samples were diluted with Annexin binding buffer to a volume of 500 μl and transferred to Trucount tubes^®^. These tubes contain a defined number of beads (2 μm in size), which allows calculation of the EV concentration by the following formula: (otal EV number in sample) = (EV count) × (total bead number)/(bead count).

### Flow cytometric analysis

Samples were analyzed in a Navios Flow Cytometer (3L10C, Beckman Coulter) using the Kaluza software and high-sensitivity settings with low flow rate, high-sensitivity settings without threshold and event trigger on side scatter. Logarithmic amplification was used for all channels and recommended isotype controls were used as negative controls. EV were gated by size (<1 μm using standard EV) in an FSC/SSC setting, by Annexin V-Cy5 positivity and antigen expression including channel compensation as previously described [22]. Measurement was stopped when at least 500 Trucount beads^®^ had been acquired.

The following combinations of antibodies were used:

- Annexin V-Cy5 + CD269/BCMA-VioBright-FITC + CD38-PE + CD27-PC5 + CD138-PacificBlue
- Annexin V-Cy5 + CD269/BCMA-VioBright-FITC + CD38-PE + CD56-PC5 + CD138-PacificBlue
- Annexin V-Cy5 + CD23-FITC + CD38-PE + CD27-PC5 + CD138-PacificBlue
- Annexin V-Cy5 + CD269/BCMA-VioBright-FITC + CD235a-PE (EVs from red blood cells = RBC-EVs).

### Statistics

Statistical analysis was carried out using IBM SPSS statistics 28.0 software.

Best response (CR, PR, MR, SD, PD) to Belantamab therapy was assessed according to IMWG criteria [23] without bone marrow histology.

Mann-Whitney-U test was used for comparison of EV counts in myeloma patients and healthy donors and Wilcoxon test for comparison of EV counts at start of therapy and at maximal level.

Spearman rank test was used to find correlations between BCMA-EV levels and free light chain (FLC) ratio, LDH levels and other treatment parameters.

Median progression and overall survival times were estimated by Kaplan-Meier analysis and univariate cox regression was used to find parameters associated with survival.

## Abbreviations

EV: extracellular vesicles
BCMA: B cell maturation antigen
ADC: antibody-drug conjugate
MMAF: monomethyl auristatin F
FLC: free light chains
RBC: red blood cells
GSI: γ-secretase inhibitor
